# The V-Sign: A Simple Radiographic Sign of Shoulder Subluxation

**DOI:** 10.7759/cureus.6501

**Published:** 2019-12-29

**Authors:** Bradley Schoch, Adam Smitherman, Mary Beth Horodyski, Aimee Struk, Joseph J King, Kevin W Farmer, Thomas Wright

**Affiliations:** 1 Orthopaedic Surgery, Mayo Clinic, Jacksonville, USA; 2 Orthopaedics and Rehabilitation, University of Florida, Gainesville, USA; 3 Orthopaedics, University of Florida, Gainesville, USA

**Keywords:** v-sign, subluxation, shoulder, radiographic, x-ray, sign, screening

## Abstract

Introduction

Shoulder subluxation is a common finding associated with orthopedic pathology. This study assesses the inter- and intra-observer reliability of a new radiographic sign used to identify glenohumeral subluxation.

Methods

Shoulders of 55 consecutive patients presenting with shoulder pain were reviewed for the presence of a “V-sign”. Three shoulder surgeons reviewed all radiographs at three separate time periods in a randomized fashion. Inter- and intra-observer reliabilities were calculated.

Results

The V-sign was identified in 26 (47%) shoulders. Intra-rater reliability was satisfactory for all the three surgeons, with kappa values of 0.85, 0.78, and 0.77, respectively. Inter-rater reliability was similarly satisfactory, with a value of 0.71. The surgeons demonstrated 100% agreement on the direction of subluxation when a V-sign was documented.

Discussion

The V-sign is a reproducible radiographic sign that can be used to detect glenohumeral subluxation in patients presenting with shoulder pain.

## Introduction

Subluxation is commonly associated with orthopedic pathology of the shoulder [1-5]. Plain radiographs are often used as an initial screening tool in the assessment of shoulder pain. Subluxation, when present, is classified based on the direction of humeral head translation in reference to the glenoid. Some authors have offered classifications based on the severity of subluxation, but no standardized measures have been widely accepted among surgeons [6].

In the non-arthritic shoulder, subluxation may be subtle, leading clinicians to delay potential evaluation and/or treatment of shoulder pathology [7]. When screening patients with shoulder pain, the presence of shoulder subluxation may be more important as a binary variable rather than the severity. Screening tests should be inexpensive, be easy to administer, inflict minimal discomfort, and be reliable and valid.

In our practice, it was noted that among patients presenting with shoulder dysfunction, radiographic signs of shoulder asymmetry were often noted. This asymmetry projects the joint space in the shape of a “V". The presence of this “V-sign” is an indication of a joint subluxation and may help identify patients who merit further workup [8]. A similar sign of subluxation has previously proven to be useful in indicating joint incongruity in proximal interphalangeal fracture-dislocations in the hand [9]. The purpose of this study is to assess the inter- and intra-observer reliabilities of this new radiographic sign of shoulder subluxation.

## Materials and methods

 Following Institutional Review Board approval, 74 consecutive patients presenting to the clinic of the senior author (Thomas Wright) with shoulder X-rays were identified. Over the same time period, a total of 850 patients were seen in the office. Radiographs were obtained in all patients at the time of presentation and included anteroposterior views with internal and external rotation and axillary lateral views. Nineteen shoulders with prior reverse shoulder arthroplasty were excluded due to the semi-constrained design of the implant and its static resistance to subluxation. Thus, 55 shoulders with radiographs of the affected shoulders were available for review.

**Figure 1:**
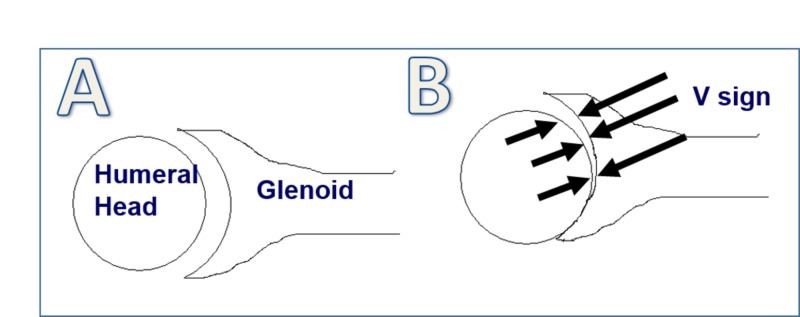
Illustration showing the normal glenohumeral articulation (A) and the presence of the V-sign (outlined by arrows) after subluxation of the humeral head in reference to the glenoid (B).

Shoulder films from all study patients were compiled, de-identified, and randomized for review. Three attending shoulder surgeons, including the senior author, reviewed all radiographs at three separate time periods. Radiographs were assessed for the “V-sign” (Figure 1). In the normal relationship of the shoulder joint, the humeral head is located concentrically across from the glenoid. However, as the humeral head subluxes into a non-concentric position, the joint space consequently appears to narrow on one side and widen on the opposite side to form the V-shape. The apex of the V, therefore, points toward the direction of subluxation. A V-sign was considered to be present if the sign was recorded on at least five of the nine review readings. When present, the direction of subluxation was also recorded.

Summary statistics were computed to describe the study sample. All analyses were performed using SAS, version 9.4 (SAS Institute Inc., Cary, NC), specifying a level of significance of 0.05. Kappa statistics were computed to measure inter- and intra-observer reliabilities.

## Results

**Figure 2:**
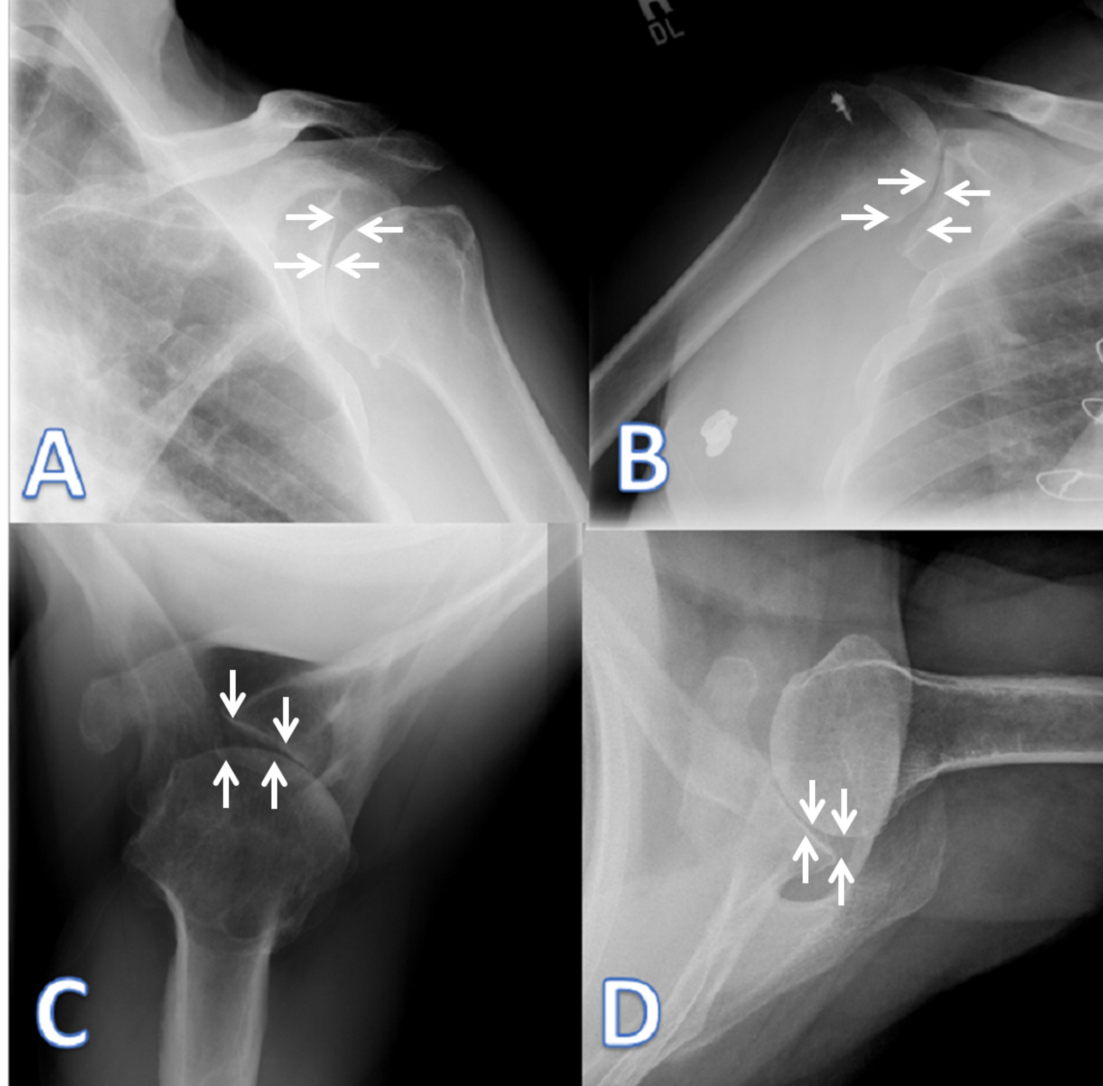
Representative examples of inferior (A), superior (B), posterior (C), and anterior (D) subluxation with the presence of the V-sign (outlined by arrows).

The mean age at evaluation for the 55 shoulders was 61.5 years (range: 31-86 years). Patient X-rays were evaluated by three attending orthopedic surgeons on three separate occasions two weeks apart in a blinded manner. The V-sign was identified in 26 (47%) shoulders. The presenting diagnoses between those with and without a V-sign were distributed similarly (see Table 1 for full details). The direction of subluxation was recorded as superior (12 [46%]), inferior (6 [23%]), posterior (4 [15%]), and anterior (4 [15%]) (Figure 2).

**Table 1:** Clinical diagnoses of patients presenting with and without a V-sign

Diagnosis	V-Sign Present	V-Sign Absent
Rotator cuff	13	15
Arthritis	4	5
Instability	1	1
Fracture	2	1
Arthroplasty	5	6
Frozen/stiff/spasticity	1	1

Intra-rater reliability was satisfactory for all the three surgeons, with kappa values of 0.85, 0.78, and 0.77, respectively. Inter-rater reliability was similarly satisfactory, with a value of 0.71. Surgeons demonstrated 100% agreement on the direction of subluxation when a V-sign was documented.

## Discussion

Clinically effective screening tests are inexpensive, are easy to administer, inflict minimal discomfort, and are reliable and valid. Radiographs remain the preferred screening mechanism for shoulder pain due to their relatively low cost and availability in many clinical office settings. This study represents the first step in the process of validating the V-sign as a clinical tool to screen for potential shoulder pathology, with the goal of identifying patients who would benefit from advanced imaging and specialty referral. Based on our results, the V-sign is a reliable radiographic sign that is reproducibly identified over time and among surgeons.

Other reports have documented that subluxation leads to an asymmetric glenohumeral joint space. While these reports have suggested that subluxation occurs opposite the area of joint space widening, this has never been validated [7,10]. In this study, agreement among reviewers in regard to the direction of subluxation was 100%, indicating that the V-sign can also be used to identify the direction of subluxation, which may be beneficial in cases of subtle change.

The next step towards validating the V-sign will be to assess its role in predicting shoulder pathology early in the disease state. Prior clinical studies have documented the presence of posterior subluxation early in the setting of primary osteoarthritis in young patients [1]. Similarly, acromiohumeral narrowing has been associated with the presence of a superior rotator cuff tear [11]. However, early subluxation of the shoulder joint has not been similarly assessed as an early sign of rotator cuff tearing. Larger prospective studies are needed to identify the ability of the V-sign to predict operative intervention and its predictive quality for various diagnoses.

This study has multiple limitations, including its retrospective nature. Rotator cuff tears represented 51% of the clinical diagnoses, potentially biasing the reliability of the V-sign for other diagnoses. However, we feel this is less likely due to the fact that subluxation is well document in both primary osteoarthritis and shoulder instability, which were also included in this study [7,10]. Patients were also selected from a surgical practice, leading to selection bias and the inability to assess the predictive nature of the V-sign in relation to operative intervention. Similarly, our patient population consisted mainly of older individuals with rotator cuff and degenerative joint disease, thus limiting the ability to generalize these findings in a younger population.

## Conclusions

The V-sign is a reproducible radiographic sign that can be used to detect shoulder subluxation in patients presenting with shoulder pain. Further study is needed to examine its role in predicting pathology on advanced imaging and its association with surgical intervention.
